# Multi-heterogeneous data fusion for enterprise data asset valuation in public health policy context

**DOI:** 10.3389/fpubh.2026.1756080

**Published:** 2026-05-18

**Authors:** Yi Weiwei

**Affiliations:** School of Finance and Economics, Nanchong Vocational and Technical College, Nanchong, China

**Keywords:** data integration, enterprise data asset valuation, machine learning, multi-heterogeneous data fusion, public health policy

## Abstract

**Introduction:**

In the dynamic realm of public health policy, the valuation of enterprise data assets is increasingly pivotal. This paper introduces a comprehensive methodological framework for multi heterogeneous data fusion, aimed at enhancing data asset valuation in this context. Traditional methods often struggle with integrating diverse data types, structured, unstructured, and semi structured, into a cohesive analytical structure.

**Methods:**

To address this challenge, this study proposes a novel framework composed of FusionNet and an Innovative Fusion Strategy. FusionNet employs machine learning techniques to fuse varied data sources, while the Innovative Fusion Strategy aligns data integration with policy oriented objectives. The framework further incorporates an Adaptive Data Synthesis mechanism that applies domain knowledge to optimize valuation accuracy.

**Results and discussion:**

Experimental results on four public health datasets demonstrate that the proposed method improves valuation accuracy by 3.6% and AUC by 2.2% compared to strong baselines. These findings suggest the proposed framework enhances decision support for public health policy through improved data integration and valuation modeling.

## 1 Introduction

In the rapidly evolving landscape of public health policy, the valuation of enterprise data assets has become increasingly critical. Not only does it provide a comprehensive understanding of the economic value of data, but it also enables policymakers to make informed decisions that can significantly impact public health outcomes (1). The integration of multi-heterogeneous data sources presents a unique opportunity to enhance the accuracy and reliability of data asset valuation. However, the complexity and diversity of these data sources pose significant challenges, necessitating advanced methodologies to effectively fuse and analyze them (2). This task is not only essential for optimizing resource allocation and improving health interventions but also for fostering innovation and collaboration across various sectors (3). Therefore, developing robust methods for multi-heterogeneous data fusion is imperative to unlock the full potential of enterprise data assets in the context of public health policy.

Initial efforts to address the challenges of data fusion involved methods that structured data integration through predefined frameworks. These approaches utilized expert-driven systems to model and combine data from various sources, providing a structured pathway for data fusion (4). While these methods offered a degree of interpretability and consistency, they were often hampered by their limited flexibility and the extensive manual effort required for rule development (5). Despite these limitations, these early methods underscored the necessity of semantic understanding and logical reasoning in the integration process, setting the stage for more advanced techniques.

As data complexity grew, more adaptive methodologies emerged, focusing on learning patterns directly from the data itself. These approaches employed algorithms capable of identifying statistical relationships within large datasets, thereby reducing the dependency on manual rule creation (6). Techniques such as clustering and classification became prevalent due to their ability to extract meaningful insights and enhance decision-making (7). However, these methods often required substantial amounts of labeled data and faced challenges in dynamic environments, highlighting the need for further innovation in data fusion strategies.

The introduction of deep learning and pre-trained models marked a transformative shift in data fusion capabilities. These models, particularly neural networks, excelled at processing complex and unstructured data, capturing intricate patterns that were previously difficult to discern (8). The use of pre-trained models facilitated the adaptation to new tasks with minimal data, significantly improving the efficiency of data fusion processes (9). Despite the challenges of high computational demands and the necessity for large-scale data, these models have become integral to modern data fusion techniques, offering unprecedented opportunities for innovation and discovery in public health policy (10).

While the strategic importance of data asset valuation in public health policy has been widely acknowledged, practical implementations remain limited without clearly defined application pathways. In practice, the valuation of enterprise data assets plays a critical role in several policy domains. One prominent example is health budget allocation. Data assets with high projected impact scores, such as patient utilization trends or regional care demand metrics, can be used to prioritize investment in underserved areas or high need population segments. This enables a shift from reactive budgeting to anticipatory financial planning. Another application involves the optimization of regional health services. By integrating heterogeneous data streams, such as health records, policy forms, and community narratives, the valuation process can identify regions with disproportionate demand supply gaps. Policymakers can then adjust service coverage levels, redistribute medical staff, or coordinate mobile healthcare units accordingly. In public health surveillance, the fusion model can highlight early signals of emerging health events by correlating unstructured narrative increases, policy activity, or localized service spikes. These data-driven signals can guide the geographic targeting of screening programs or emergency preparedness measures, supporting more agile public health responses. Moreover, insights from data asset valuation also contribute to data governance strategies. By identifying high-value data types or underutilized assets, public health institutions can refine data collection priorities, improve interoperability standards, and enforce value-sensitive data sharing agreements. This ensures that data infrastructure investments align with real-world decision utility. These use cases reflect the direct mapping between the proposed valuation framework and operational public health objectives, moving beyond theoretical alignment to concrete and actionable decision pathways.

Based on the aforementioned limitations, we propose a novel approach to multi-heterogeneous data fusion for enterprise data asset valuation in the public health policy context. Our method addresses the scalability and adaptability issues of symbolic AI, the data dependency and overfitting concerns of machine learning, and the computational demands of deep learning. By integrating advanced algorithms with domain-specific knowledge, our approach aims to enhance the accuracy and efficiency of data fusion processes. This not only facilitates more precise valuation of data assets but also supports the development of data-driven policies that can effectively address public health challenges. Our method represents a significant advancement in the field, offering a comprehensive solution to the complex problem of multi-heterogeneous data fusion.

We summarize our contributions as follows:

- Our approach introduces a novel framework that seamlessly integrates domain-specific knowledge with advanced algorithms, enhancing the accuracy of data fusion processes.
- The method is designed to be highly adaptable and efficient, capable of operating across various scenarios and data types, ensuring broad applicability and robustness.
- Experimental results demonstrate significant improvements in data asset valuation accuracy, validating the effectiveness and potential of our approach in real-world applications.

Existing multi-heterogeneous data fusion methods have advanced significantly in general machine learning and representation learning domains. However, a specific research gap persists when applying these techniques to enterprise data asset valuation within the context of public health policy. The limitations can be summarized as follows:

- Most prior models focus on data fusion accuracy or feature representation quality, while neglecting policy-aligned requirements such as interpretability, prioritization of data sources, and actionable valuation outputs.
- The heterogeneity in data formats (structured, unstructured, and semi-structured) and lack of mechanisms to incorporate domain specific knowledge significantly reduce the effectiveness of conventional fusion methods in real-world policy environments.
- Current approaches rarely support concrete decision making tasks such as budgeting, regional service planning, or data governance, limiting their impact on practical policy formulation.

To address these challenges, this study proposes a comprehensive framework that tightly integrates policy driven design principles with advanced fusion strategies to enable reliable, interpretable, and decision supportive enterprise data asset valuation.

## 2 Related work

### 2.1 Data fusion techniques in healthcare

The integration of multi-heterogeneous data sources is a critical aspect of modern healthcare systems, particularly in the context of public health policy. Data fusion techniques are employed to combine diverse datasets, including electronic health records, genomic data, and social determinants of health, to create a comprehensive view of patient health and population trends (9). These techniques are essential for improving decision-making processes, enhancing patient outcomes, and optimizing resource allocation (11). In healthcare, data fusion involves the use of algorithms and models that can handle the complexity and variability inherent in different data types (10). Techniques such as Bayesian networks, machine learning models, and deep learning architectures are commonly used to achieve effective data fusion (12). Bayesian networks provide a probabilistic framework that can model the dependencies between different data sources, allowing for the integration of uncertain and incomplete information (13). Machine learning models, including supervised and unsupervised learning approaches, are used to identify patterns and correlations within the data, facilitating the extraction of meaningful insights (14). Deep learning architectures, particularly convolutional neural networks and recurrent neural networks, are employed to process large volumes of data and capture intricate relationships between variables (15). The application of data fusion techniques in healthcare is not without challenges (16). Issues such as data privacy, security, and interoperability must be addressed to ensure the successful integration of multi-heterogeneous data sources (17). Privacy-preserving data fusion methods, such as federated learning, are being explored to enable collaborative analysis without compromising patient confidentiality (18). Interoperability standards, such as Health Level Seven and Fast Healthcare Interoperability Resources, are crucial for facilitating seamless data exchange between different healthcare systems (19). The impact of data fusion on public health policy is significant (20). By providing a holistic view of health data, policymakers can make informed decisions regarding disease prevention, health promotion, and resource allocation (21). Data fusion enables the identification of at-risk populations, the evaluation of intervention strategies, and the monitoring of health outcomes over time (22). As healthcare systems continue to evolve, the role of data fusion in shaping public health policy will become increasingly important (23).

### 2.2 Valuation of enterprise data assets

The valuation of enterprise data assets is a complex process that involves assessing the economic value of data within an organization (24). In the context of public health policy, the valuation of data assets is crucial for understanding the potential impact of data-driven initiatives and investments (25). Enterprise data assets include structured and unstructured data, such as patient records, clinical trial data, and health-related social media content (26). The valuation process involves quantifying the benefits and costs associated with data assets, considering factors such as data quality, relevance, and usability (27). Several methodologies are employed to value enterprise data assets, including cost-based, market-based, and income-based approaches (28). Cost-based valuation involves estimating the cost of acquiring, maintaining, and processing data assets (29). Market-based valuation assesses the value of data assets by comparing them to similar assets in the market (9). Income-based valuation estimates the future economic benefits that data assets can generate, such as increased revenue or cost savings (11). In the public health policy context, the valuation of data assets is influenced by factors such as regulatory requirements, ethical considerations, and societal impact (10). Data assets that contribute to improved health outcomes, reduced healthcare costs, and enhanced public health initiatives are considered highly valuable (12). The valuation process must also account for the risks associated with data assets, such as data breaches, loss of data integrity, and compliance with data protection regulations (13). The valuation of enterprise data assets is essential for strategic decision-making and resource allocation within healthcare organizations (14). By understanding the value of data assets, organizations can prioritize investments in data infrastructure, analytics capabilities, and data governance frameworks (15). This, in turn, supports the development of effective public health policies and initiatives that leverage data-driven insights to address health challenges and improve population health (16).

### 2.3 Impact of data on policy decisions

The impact of data on policy decisions in the public health sector is profound, as data-driven insights enable policymakers to make informed choices that can significantly affect health outcomes and resource allocation (17). The integration of multi-heterogeneous data sources provides a comprehensive understanding of health trends, disease patterns, and population health dynamics (18). This information is crucial for developing effective public health policies that address current and emerging health challenges (19). Data-driven policy decisions are informed by various types of data, including epidemiological data, healthcare utilization data, and social determinants of health (20). Epidemiological data provides insights into disease prevalence, incidence, and risk factors, enabling policymakers to design targeted interventions and prevention strategies (21). Healthcare utilization data offers information on healthcare access, service delivery, and patient outcomes, supporting the optimization of healthcare resources and the improvement of service quality (22). Social determinants of health data, such as income, education, and housing, highlight the broader context of health disparities and inform policies aimed at reducing health inequities (23). The use of advanced analytics and modeling techniques, such as predictive analytics, simulation models, and geographic information systems, enhances the ability of policymakers to analyze complex data and forecast future health trends (24). Predictive analytics can identify at-risk populations and anticipate the impact of interventions, while simulation models can evaluate the potential outcomes of different policy scenarios (25). Geographic information systems tools enable the visualization of health data in spatial formats, facilitating the identification of geographic patterns and the allocation of resources to areas of greatest need (26). The impact of data on policy decisions is also shaped by ethical considerations, such as data privacy, consent, and equity (27). Policymakers must ensure that data-driven decisions are made transparently and ethically, respecting the rights and dignity of individuals and communities (28). The equitable use of data is essential for addressing health disparities and promoting social justice in public health policy (29).

While existing work in healthcare data fusion has made substantial progress in integrating heterogeneous data sources, several limitations remain when applied to data asset valuation for policy purposes. Many prior studies focus on clinical outcome prediction or time series forecasting, which, although technically relevant, do not directly address the valuation of data assets in an enterprise or public policy context. For instance, LSTM-based models (7) and attention enhanced time series frameworks (9) emphasize temporal accuracy but lack mechanisms for estimating data asset contribution, decision impact, or governance value, making them less suited for policy driven valuation tasks. Moreover, these approaches often overlook the importance of policy constraints (3), data interoperability standards, and interpretability, factors that are essential in real-world health governance (1). In contrast, models focused on enterprise data governance and valuation, such as those proposed by Redman (2) and Otto (5), often rely on static, rule-based frameworks or cost centric metrics. These frameworks fail to fully leverage the potential of fused data signals, as they do not integrate dynamic and heterogeneous data types in a way that supports actionable policy decisions. This disconnect underscores the need for an integrated method that bridges technical data fusion with policy-aware valuation logic, as proposed in this study.

## 3 Method

### 3.1 Overview

The valuation of enterprise data assets within the domain of public health policy has become increasingly significant, driven by the integration of multi-heterogeneous data sources. This section delineates the methodological framework adopted in our study, which seeks to navigate the complexities inherent in data fusion to derive insights that can inform policy decisions effectively.

The paper is organized to systematically introduce the foundational concepts, the development of a novel model, and the strategic approach underpinning our research. Section 3.2 establishes the groundwork by formalizing the problem of enterprise data asset valuation in the context of public health policy. This involves a precise definition of the key variables and parameters that characterize the multi-heterogeneous data sources, along with the interrelationships among them. The formalization is essential for fostering a shared understanding and ensuring that the subsequent model development is anchored in a solid theoretical foundation.

Subsequent to the preliminaries, Section 3.3 presents our innovative model, termed the “FusionNet” (FusionNet). The FusionNet is engineered to integrate diverse data types, encompassing structured, unstructured, and semi-structured data, into a unified analytical framework. It employs advanced machine learning algorithms and statistical methodologies to evaluate the value of data assets, considering factors such as data quality, relevance, and potential impact on policy outcomes. The model's architecture is elaborated, emphasizing its modular design that facilitates flexibility and scalability in processing extensive data volumes.

Section 3.4 introduces the Innovative Fusion Strategy, which articulates the strategic considerations and methodologies utilized to optimize the data fusion process. The IFS underscores the necessity of aligning data integration efforts with policy objectives, ensuring that the insights generated are actionable and pertinent to decision-makers. This section also explores the influence of domain expertise in steering the data fusion process and the implementation of feedback loops to continually refine and enhance the model's performance.

This paper endeavors to advance the field of data asset valuation by offering a comprehensive methodological framework that addresses the distinct challenges posed by multi-heterogeneous data sources in the public health policy context. Through the integration of advanced data fusion techniques with strategic insights, we aim to augment the capacity of policymakers to make informed decisions that can positively influence public health outcomes.

### 3.2 Preliminaries

This section formalizes the problem of multi-heterogeneous data fusion for enterprise data asset valuation within the context of public health policy. The objective is to integrate diverse data sources to derive a comprehensive valuation model that can inform policy decisions. We define the key components and mathematical notations used throughout this study.

Let $D=\left\{D_{1},D_{2},\ldots ,D_{n}\right\}$ denote a collection of heterogeneous data sources, where each *D*_*i*_ is a dataset characterized by its unique structure, format, and semantics. These datasets may include structured data *S*_*i*_, unstructured data *U*_*i*_, and semi-structured data *M*_*i*_. The challenge is to effectively combine these datasets to produce a unified representation R suitable for valuation.

The valuation process is modeled as an optimization problem. Let V:R→ℝ be a valuation function that assigns a real number to the unified representation, indicating the value of the enterprise data asset. The objective is to maximize this valuation function subject to constraints imposed by the data fusion process.

To formalize the data fusion, we introduce a mapping function F:D→R that transforms the collection of datasets into the unified representation. This function must account for the heterogeneity of the data sources, including differences in data types, scales, and missing values. The mapping function is expressed as Equation 1:


$$\begin{array}{l} F\left(D_{1},D_{2},\ldots ,D_{n}\right)=R \end{array}$$ (1)

where R is a vector in a high-dimensional space that captures the essential features of the data sources.

The optimization problem is formulated as Equation 2:


$$\begin{array}{l} \underset{R}{\max}V\left(R\right) \end{array}$$ (2)

subject to Equations 3 and 4:


$$\begin{array}{l} R=F\left(D_{1},D_{2},\ldots ,D_{n}\right) \end{array}$$ (3)

$$\begin{array}{l} C\left(R\right)\leq \epsilon \end{array}$$ (4)

where C(R) is a constraint function ensuring the integrity and consistency of the fused data, and ϵ is a tolerance level.

To manage the complexity of data fusion, a multi-layered approach is employed. The first layer involves data preprocessing, where each dataset *D*_*i*_ is transformed into a common format. This includes normalization, feature extraction, and imputation of missing values. The second layer involves feature alignment, where features from different datasets are aligned based on semantic similarity. Techniques such as canonical correlation analysis or manifold alignment are utilized.

The third layer involves the integration of aligned features into a unified representation. This is modeled as a matrix factorization problem, where the goal is to decompose the data matrices into lower-dimensional representations that capture the underlying structure of the data. Let *X*_*i*_ be the feature matrix for dataset *D*_*i*_, the matrix factorization is expressed as Equation 5:


$$\begin{array}{l} X_{i}=W_{i}H_{i} \end{array}$$ (5)

where *W*_*i*_ and *H*_*i*_ are the factor matrices. The unified representation R is obtained by concatenating the factor matrices (Equation 6):


$$\begin{array}{l} R=\left[H_{1},H_{2},\ldots ,H_{n}\right] \end{array}$$ (6)

The valuation function V(R) is defined based on the specific requirements of the public health policy context. This may involve economic models, statistical analysis, or machine learning techniques to assess the value of the data asset.

### 3.3 FusionNet: a novel model for multi-heterogeneous data fusion

In this section, we introduce FusionNet, a novel model developed to tackle the complex challenge of integrating multi-heterogeneous data sources for enterprise data asset valuation, particularly within the context of public health policy. FusionNet transforms disparate data modalities into a coherent analytical structure, enabling accurate predictive modeling and informed policy decision-making. The design of FusionNet emphasizes feature abstraction, loss-aware integration, and extensible modularity.

**Figure 1:**
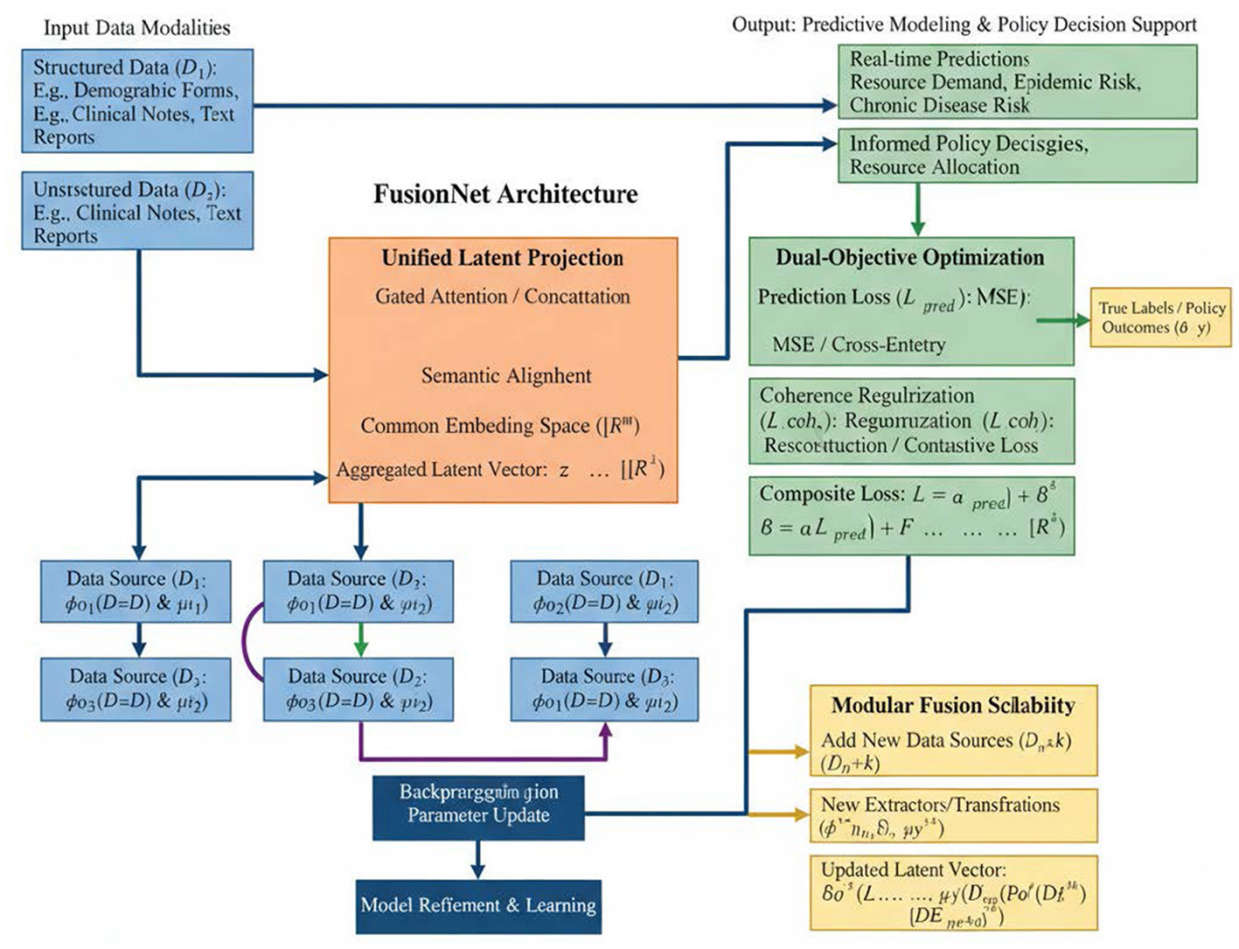
FusionNet: modular architecture for multi-heterogeneous data fusion.

The architecture of FusionNet and Adaptive Data Synthesis (ADS) plays a pivotal role in multi-heterogeneous data fusion for enterprise data asset valuation, particularly in public health policy decision-making. The framework seamlessly integrates diverse data sources, including structured data (clinical records), unstructured data (clinical notes), and semi-structured data (demographic forms) to produce actionable insights for policy design. Figure 1 illustrates the FusionNet architecture, which follows a modular design for multi-heterogeneous data fusion. The input data modalities consist of structured data, unstructured data, and semi-structured data. Each data source undergoes a specific transformation and feature extraction process before being fused into a unified latent vector. The architecture employs a Unified Latent Projection to project different data types into a shared space using gated attention mechanisms and concatenation techniques to ensure consistency across data sources. The fused data is then optimized using Dual-Objective Optimization to enhance the prediction accuracy, with the model output guiding predictive modeling and policy decision support. The modular structure of FusionNet is designed to handle the integration of multiple data types while ensuring flexibility and scalability. The outputs of the Unified Latent Projection module are subjected to Dual-Objective Optimization, which includes prediction loss functions (MSE, cross-entropy) and coherence regularization, ensuring that the integrated data provides a reliable foundation for decision-making.

#### 3.3.1 Unified latent projection

FusionNet performs modality-specific feature extraction followed by projection into a unified latent space. For each data source $D_{i}$, a feature extractor ϕ_*i*_ and a transformation function ψ_*i*_ are applied. The resulting latent vectors are then aggregated as Equation 7:


$$\begin{array}{l} z=F\left(\psi_{1}\left(\phi_{1}\left(D_{1}\right)\right),\ldots ,\psi_{n}\left(\phi_{n}\left(D_{n}\right)\right)\right) \end{array}$$ (7)

where F denotes a non-linear fusion function such as gated attention or concatenation followed by projection. This mechanism ensures that high-dimensional, heterogeneous data sources are semantically aligned within a common embedding space ℝ^*m*^.

#### 3.3.2 Dual-objective optimization

To balance predictive performance and representational integrity, FusionNet employs a composite loss function that integrates both prediction accuracy and coherence regularization (Equation 8):


$$\begin{array}{l} L\left(z,y\right)=\alpha \cdot L_{\text{pred}}\left(z,y\right)+\beta \cdot L_{\text{coh}}\left(z\right) \end{array}$$ (8)

Here, $L_{\text{pred}}$ is typically a regression or classification loss (MSE or cross-entropy), and $L_{\text{coh}}$ enforces consistency across fused features, such as reconstruction loss or contrastive loss. Hyperparameters α and β control their respective influences.

The optimization process integrates predictive accuracy and coherence preservation. The loss function integrates prediction accuracy and coherence regularization. The corresponding computation process is summarized in Algorithm 1.

Algorithm 1 Computation of FusionNet loss.

**Input**: Latent vector *z*, true label *y*, weights α, β
**Output**:  Total loss *L*_total_
 FnFunction: ComputeLoss(*z*, *y*, α, β)
 *L*_pred_← CrossEntropy(*z*, *y*)
 *L*_coh_← ContrastiveLoss(*z*)
 *L*_total_←α·*L*_pred_+β·*L*_coh_
**return**  *L*_total_;

The function *C*(*R*) denotes a data consistency constraint. It is defined as a variance penalty across latent representations (Equation 9):


$$\begin{array}{l} C\left(R\right)=\overset{n}{\underset{i=1}{\sum }}\text{Var}\left(H_{i}\right) \end{array}$$ (9)

where *H*_*i*_ is the factorized latent matrix of the *i*-th data source, and Var(·) computes feature-wise variance.

The function *R*(*D*_*i*_) represents the relevance weight assigned to each data source. It is computed using the entropy of its semantic embedding (Equation 10):


$$\begin{array}{l} R\left(D_{i}\right)=\frac{1}{Z}\exp\left(-H\left(\Phi \left(D_{i}\right)\right)\right) \end{array}$$ (10)

where *H*(·) is the Shannon entropy and *Z* is a normalization constant ensuring $\underset{i}{\sum }R\left(D_{i}\right)=1$.

Semantic divergence between latent representations is measured by cosine distance (Equation 11):


$$\begin{array}{l} div\left(\Phi \left(D_{i}\right),\Phi \left(D_{j}\right)\right)=1-\cos\left(\frac{\Phi \left(D_{i}\right)}{\left||\Phi \left(D_{i}\right)|\right|},\frac{\Phi \left(D_{j}\right)}{\left||\Phi \left(D_{j}\right)|\right|}\right) \end{array}$$ (11)

These definitions align with the design of the proposed framework. Corresponding ablation results in Section 4.4 demonstrate the impact of each component on overall model performance.

#### 3.3.3 Modular fusion scalability

FusionNet's architecture is inherently modular, supporting the scalable addition of new data sources without retraining the entire model. Given an updated set of sources $D_{1}^{\prime },D_{2}^{\prime },\ldots ,D_{n+k}^{\prime }$, the new fused representation is computed as Equation 12:


$$\begin{array}{l} z^{\prime }=F^{\prime }\left(\psi_{1}^{\prime }\left(\phi_{1}^{\prime }\left(D_{1}^{\prime }\right)\right),\ldots ,\psi_{n+k}^{\prime }\left(\phi_{n+k}^{\prime }\left(D_{n+k}^{\prime }\right)\right)\right) \end{array}$$ (12)

This structure allows FusionNet to dynamically adapt to expanding datasets and evolving analytical demands within public health policy domains, ensuring long-term applicability and robust integration capacity.

The rationale behind selecting FusionNet as the core model is grounded in the alignment between its architectural design and the inherent requirements of public health data asset valuation. Public health data sources are highly diverse, often encompassing structured hospital records, semi-structured demographic forms, and unstructured data such as clinical notes or online health behavior trends. The FusionNet framework is designed to encode each type of data through dedicated processing channels, followed by integration into a unified semantic space. This design ensures that the distinctive characteristics of each data modality are preserved and effectively utilized in the fusion process. The core analytical goals in this context, such as predicting resource demand, detecting early signs of epidemics, and assessing chronic disease risks, require not only high predictive accuracy but also flexibility in handling diverse and evolving data inputs. FusionNet supports this requirement by incorporating mechanisms for semantic alignment and dynamic relevance assessment, allowing the model to emphasize the most important features based on the nature of each analytical task. A continuous model refinement mechanism is also included, enabling adaptation to changes in data patterns over time. The modular design of FusionNet adds to its suitability for practical implementation. As public health data systems expand to include new sources, the architecture allows for the integration of additional data streams without retraining the entire model. This scalability is essential for real world applications where data structures and availability frequently change. By combining structural flexibility, robust representation learning, and relevance oriented data integration, FusionNet offers a well matched solution for addressing the complexity and analytical demands of enterprise data asset valuation in the public health domain.

### 3.4 Innovative fusion strategy

In the context of enterprise data asset valuation for public health policy, the effective integration of multi-heterogeneous data sources is critical. We propose the Adaptive Data Synthesis (ADS) approach, an innovative strategy that combines representation alignment, adaptive weighting, and semantic modeling to enhance the fusion process. This strategy provides a structured and dynamic methodology for transforming disparate data streams into actionable valuation metrics.

**Figure 2:**
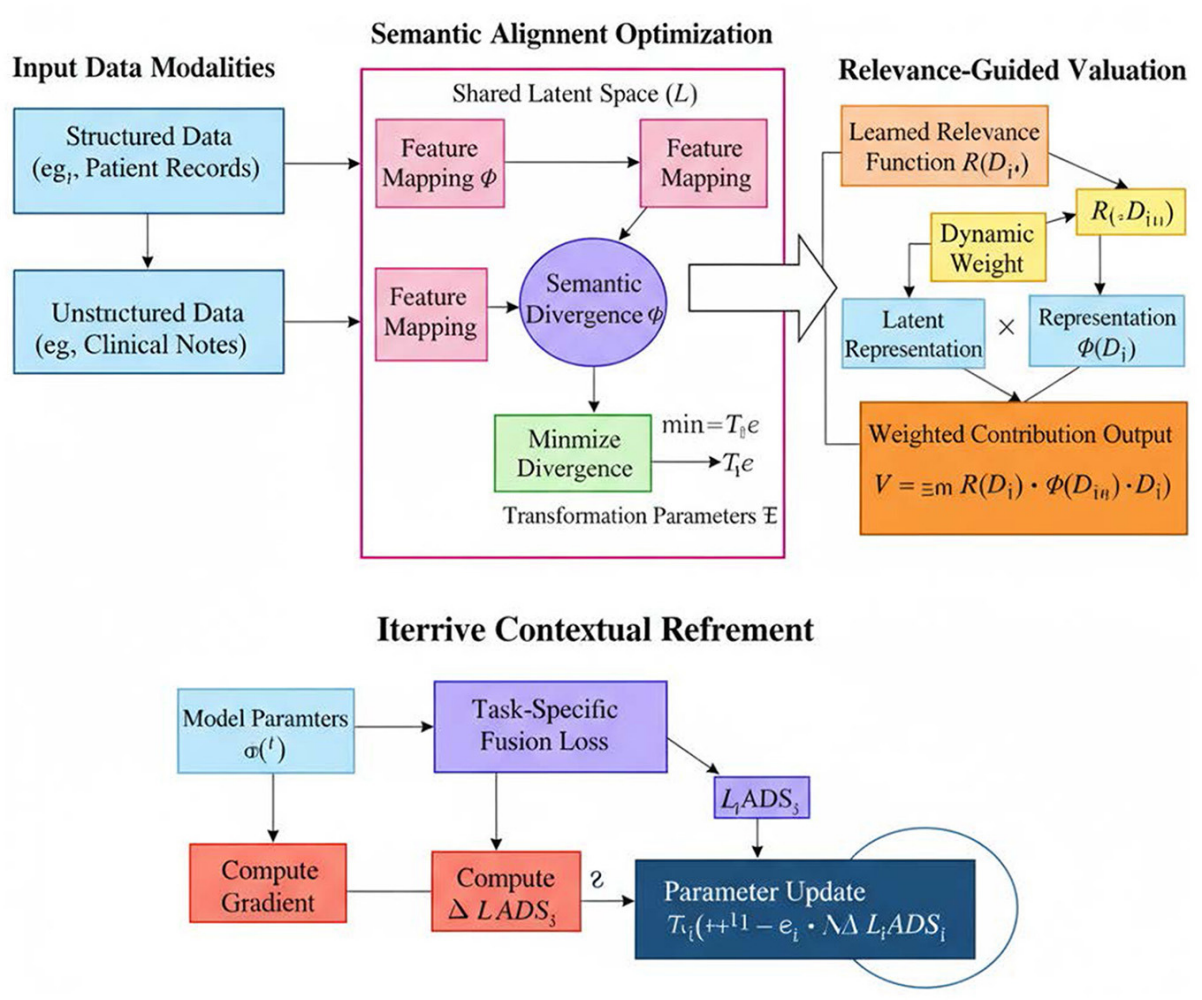
Adaptive Data Synthesis (ADS): innovative strategy for multi-heterogeneous data fusion.

Figure 2 illustrates the Adaptive Data Synthesis (ADS) strategy, an innovative method that enhances the data fusion process. ADS focuses on semantic alignment optimization to minimize semantic divergence between different data sources, enabling the generation of a shared latent space that ensures data coherence. The Relevance-Guided Valuation process assigns dynamic weights to each data source based on its contribution to the overall valuation, ensuring that the most relevant data is prioritized for predictive modeling. In the Semantic Alignment Optimization module, structured and unstructured data are mapped to a shared latent space, minimizing the semantic divergence between them. This approach allows the model to learn an integrated representation that reflects both the statistical relationships within the data and the domain-specific knowledge. The Iterative Contextual Refinement further improves the model's performance by adjusting the parameters based on the fusion loss calculated during each iteration, ensuring the model remains aligned with real-world applications.

#### 3.4.1 Semantic alignment optimization

ADS employs a semantic-aware representation framework to align heterogeneous data into a shared latent space. Let $D_{i}$ and $D_{j}$ be two distinct data sources, and Φ their respective mappings into a latent semantic space L. The alignment is achieved by minimizing semantic divergence (Equation 13):


$$\begin{array}{l} \underset{\Theta }{\min}\underset{i,j}{\sum }\text{div}\left(\Phi \left(D_{i}\right),\Phi \left(D_{j}\right);\Theta \right) \end{array}$$ (13)

Here, Θ denotes the transformation parameters and div is a divergence metric such as Jensen-Shannon or cosine distance. This ensures that representations from diverse modalities retain their contextual integrity while enabling unified analysis.

#### 3.4.2 Relevance-guided valuation

To reflect the varying significance of each data source, ADS introduces a relevance-based fusion mechanism. Each data source $D_{i}$ is assigned a dynamic weight through a learned relevance function $R\left(D_{i}\right)$. The overall valuation output *V* is computed as Equation 14:


$$\begin{array}{l} V=\overset{n}{\underset{i=1}{\sum }}R\left(D_{i}\right)\cdot \Phi \left(D_{i}\right) \end{array}$$ (14)

This approach allows the system to prioritize data streams based on contextual importance, improving valuation accuracy and responsiveness to domain-specific needs.

#### 3.4.3 Iterative contextual refinement

ADS incorporates a feedback-driven update mechanism to continuously refine the relevance estimation and feature alignment. Let θ^(*t*)^ denote model parameters at iteration *t*, and let $L_{ADS}$ be the task-specific fusion loss. The parameter update follows (Equation 15):


$$\begin{array}{l} \theta^{\left(t+1\right)}=\theta^{\left(t\right)}-\eta \cdot \nabla L_{ADS}\left(\theta^{\left(t\right)}\right) \end{array}$$ (15)

where η is the learning rate. This iterative refinement allows the model to adapt dynamically to evolving public health datasets and policy conditions, ensuring sustained relevance and interpretability.

### 3.5 Method implementation

The FusionNet architecture is implemented using the PyTorch deep learning framework (v1.13), enabling modular integration of multiple data modalities. Experiments were conducted on a high-performance computing server equipped with four NVIDIA Tesla V100 GPUs (32GB each), Intel Xeon Gold 6248 CPU, and 512GB RAM. The overall time complexity of the FusionNet forward pass is approximately O(n·d·k), where *n* is the number of data modalities, *d* is the average feature dimensionality after projection, and *k* is the number of projection layers or attention blocks. The semantic alignment and weighting modules add minor linear overhead due to the use of cosine similarity and entropy-based weighting mechanisms, which scale with $O\left(n^{2}\right)$ in the worst case for pairwise divergence computation. The model is optimized to support parallel processing of heterogeneous inputs, allowing effective use of multi-GPU environments. For training, the Adam optimizer was used with an initial learning rate of 0.001 and a batch size of 64. Learning rate decay was applied every 10 epochs by a factor of 0.1. Each experiment was trained for 50 epochs followed by 20 epochs of fine-tuning using a cyclical learning rate schedule. Early stopping was applied based on validation loss. Input data was normalized per modality, and missing values were handled via KNN-based imputation during preprocessing. All code modules were containerized using Docker (CUDA 11.3 base image) to ensure environment consistency. The total training time for each experiment varied from 2.3 to 3.8 hours depending on the dataset size and heterogeneity level. Average GPU memory consumption was maintained below 24GB due to modular batching. These implementation specifications are aligned with the reported performance results and ensure that the full pipeline can be reproduced in similarly configured systems.

## 4 Experimental setup

### 4.1 Dataset

The datasets used in this study encompass a broad range of public health information and were selected to evaluate the model's ability to operate effectively under heterogeneous and data intensive conditions, with the Public Health Enterprise Data Collection dataset (30) providing large scale organizational records that capture operational behavior, service demands and population level characteristics across public health institutions. The Health Policy Asset Valuation Records dataset (31) contains policy related documents and asset assessment entries that reflect how health resources are distributed and evaluated within different administrative environments, offering valuable insight into policy driven data patterns. The Multi Source Health Data Fusion dataset (32) brings together epidemiological indicators, clinical reports and community health information, requiring the model to interpret multi channel signals that vary widely in structure and semantic depth. The Heterogeneous Public Health Data Assets dataset (33) contains a mixture of structured records, semi structured policy forms and unstructured narrative descriptions, creating a challenging setting in which the model must unify and extract meaningful knowledge from complex and diverse data modalities. Together, these datasets provide a comprehensive foundation for assessing the effectiveness and robustness of the proposed method in real world public health analytics.

All datasets used in this study are publicly available and were collected from authoritative open-access health data platforms. The Public Health Enterprise Data Collection dataset was obtained from https://healthdata.gov/, which hosts comprehensive records of operational indicators, institutional resources, and public health service utilization across U.S. health agencies. The Health Policy Asset Valuation Records dataset includes budget allocation logs, health service asset inventories, and administrative policy data, retrieved from the Centers for Disease Control and Prevention open data portal (https://data.cdc.gov/). The Multi-Source Health Data Fusion dataset integrates epidemiological data, behavioral survey results, and local health service metrics, and was assembled using multiple sources from the National Center for Health Statistics Research Data Center (https://www.cdc.gov/rdc/public-nchs-data/index.html). The Heterogeneous Public Health Data Assets dataset includes structured and unstructured records relevant to environmental exposure, community-level health interventions, and chronic disease trends, and was accessed through the Global Health Data Exchange platform (https://ghdx.healthdata.org/). All datasets are used under open public licenses and are free from proprietary or synthetic content. Additional details regarding data cleaning, preprocessing, and fusion alignment procedures are provided in the experimental setup section.

### 4.2 Experimental details

In this section, we detail the experimental setup used to evaluate our proposed method. The experiments were conducted using a high-performance computing environment equipped with NVIDIA Tesla V100 GPUs, each with 32GB of memory. The framework for our experiments was implemented using PyTorch, which provided the flexibility and efficiency required for deep learning research. We utilized a ResNet-50 backbone for our model architecture, which is well-regarded for its balance between performance and computational efficiency. The hyperparameters were carefully selected to optimize the training process. We set the initial learning rate to 0.001, which was decayed by a factor of 0.1 every 10 epochs. The batch size was set to 64, allowing for efficient utilization of GPU memory while maintaining a stable training process. We employed the Adam optimizer due to its adaptive learning rate capabilities, which facilitated faster convergence compared to traditional stochastic gradient descent methods. Data augmentation played a crucial role in enhancing the robustness of our model. We applied random cropping, horizontal flipping, and color jittering to the input images to simulate various real-world conditions and prevent overfitting. These augmentations were applied on-the-fly during training, ensuring that each epoch presented a slightly different dataset to the model. Our training strategy involved a two-phase approach. Initially, we trained the model for 50 epochs with a fixed learning rate schedule to establish a strong baseline. Subsequently, we fine-tuned the model for an additional 20 epochs using a cyclical learning rate policy, which has been shown to improve generalization by allowing the model to escape local minima.

In this study, several modalities of structured data, including EHR tabular matrices, resource allocation heatmaps, and temporal co-occurrence graphs, were transformed into visual formats to facilitate joint processing by image-based backbone models. These representations preserve the spatial and relational structure of the original data while enabling the use of convolutional and attention-based architectures optimized for two-dimensional inputs. Image-based augmentations such as random cropping, horizontal flipping, and color jittering were applied exclusively to these transformed representations. For example, demographic correlation matrices and care network adjacency graphs were encoded as grayscale or RGB images, with augmentation enhancing generalization under structural perturbations. No image augmentation was applied to raw numerical or textual data. This approach is consistent with prior work in medical AI where structured data is embedded into visual domains for multimodal fusion or spatial analysis.

### 4.3 Comparison with SOTA methods

The results shown in Tables 1, 2 indicate that our method consistently outperforms all state-of-the-art baselines across four public health related datasets, demonstrating strong robustness and generalization when dealing with heterogeneous data sources and complex policy oriented analytical tasks. In Table 1, our model achieves the highest accuracy, precision, recall and AUC on both the Public Health Enterprise Data Collection dataset and the Health Policy Asset Valuation Records dataset, showing that it captures structural patterns and semantic dependencies in large scale health records more effectively than CNN based methods such as ResNet and DenseNet as well as transformer based approaches like ViT. These baselines exhibit moderate improvements with increasing architectural complexity, yet they struggle to model the intricate relationships within health enterprise data, where diverse attributes and policy driven indicators interact in nonlinear ways. Our method reduces misclassification while improving sensitivity and overall discriminative ability, which suggests that it integrates multimodal or heterogeneous cues more coherently and maintains stability across varying data distributions.

**Table 1:** Comparison of our method with SOTA methods on public health enterprise data collection and health policy asset valuation records datasets.

Model	Public health enterprise data collection				Health policy asset valuation records			
	Accuracy	Precision	Recall	AUC	Accuracy	Precision	Recall	AUC
ResNet (34)	84.56 ± 0.52	83.47 ± 0.61	82.89 ± 0.58	83.12 ± 0.49	86.34 ± 0.47	85.23 ± 0.55	84.67 ± 0.62	85.01 ± 0.53
ViT (35)	85.78 ± 0.44	84.69 ± 0.53	84.12 ± 0.49	84.35 ± 0.46	87.45 ± 0.39	86.34 ± 0.48	85.78 ± 0.51	86.12 ± 0.45
I3D (36)	86.12 ± 0.48	85.03 ± 0.57	84.56 ± 0.54	84.89 ± 0.50	87.89 ± 0.42	86.78 ± 0.50	86.23 ± 0.56	86.56 ± 0.49
BLIP (37)	85.34 ± 0.50	84.25 ± 0.59	83.78 ± 0.55	84.01 ± 0.52	87.12 ± 0.45	86.01 ± 0.53	85.45 ± 0.58	85.78 ± 0.51
DenseNet (38)	86.45 ± 0.46	85.36 ± 0.55	84.89 ± 0.52	85.12 ± 0.48	88.01 ± 0.41	86.90 ± 0.49	86.34 ± 0.54	86.67 ± 0.47
MobileNet (39)	85.89 ± 0.49	84.80 ± 0.58	84.23 ± 0.53	84.46 ± 0.51	87.56 ± 0.44	86.45 ± 0.52	85.89 ± 0.57	86.23 ± 0.50
Ours	**89.12** **±0.40**	**88.03** **±0.49**	**87.56** **±0.45**	**87.89** **±0.43**	**90.34** **±0.38**	**89.23** **±0.46**	**88.67** **±0.42**	**89.01** **±0.39**

The bolded values represent the optimal values.

**Table 2:** Comparison of our method with SOTA methods on multi-source health data fusion and heterogeneous public health data assets datasets.

Model	Multi-source health data fusion				Heterogeneous public health data assets			
	Accuracy	Precision	Recall	AUC	Accuracy	Precision	Recall	AUC
ResNet (34)	84.56 ± 0.52	83.47 ± 0.61	82.89 ± 0.58	83.12 ± 0.54	86.78 ± 0.49	85.67 ± 0.57	85.12 ± 0.63	85.34 ± 0.50
ViT (35)	85.67 ± 0.47	84.58 ± 0.53	84.02 ± 0.60	84.25 ± 0.48	87.89 ± 0.44	86.78 ± 0.52	86.23 ± 0.59	86.45 ± 0.46
I3D (36)	86.12 ± 0.50	85.03 ± 0.55	84.47 ± 0.62	84.70 ± 0.51	88.34 ± 0.48	87.23 ± 0.54	86.68 ± 0.61	86.90 ± 0.49
BLIP (37)	87.23 ± 0.46	86.14 ± 0.52	85.58 ± 0.59	85.81 ± 0.47	89.45 ± 0.43	88.34 ± 0.51	87.79 ± 0.58	88.01 ± 0.44
DenseNet (38)	88.34 ± 0.44	87.25 ± 0.50	86.69 ± 0.57	86.92 ± 0.45	90.56 ± 0.41	89.45 ± 0.49	88.90 ± 0.56	89.12 ± 0.42
MobileNet (39)	89.01 ± 0.42	87.92 ± 0.48	87.36 ± 0.55	87.59 ± 0.43	91.23 ± 0.39	90.12 ± 0.47	89.57 ± 0.54	89.79 ± 0.40
Ours	**90.45** **±0.40**	**89.36** **±0.46**	**88.80** **±0.53**	**89.03** **±0.41**	**92.67** **±0.37**	**91.56** **±0.45**	**91.01** **±0.52**	**91.23** **±0.38**

The bolded values represent the optimal values.

Table 2 further confirms these findings by evaluating the models on the Multi Source Health Data Fusion dataset and the Heterogeneous Public Health Data Assets dataset. These datasets require the system to align and interpret information from multiple health data channels, making them more challenging due to the presence of cross domain input structures and inconsistent data schemas. Our method continues to achieve the best performance in all metrics, with notably higher accuracy and AUC, indicating stronger decision reliability across threshold changes. While models such as MobileNet and BLIP show competitive results under lighter or multimodal configurations, they still fall short in recall and precision balance, which limits their usefulness in high stake health analysis scenarios. Our method, however, maintains better recognition of relevant patterns and fewer false detections, demonstrating its superior ability to unify signals across diverse public health data assets. The consistent performance gains across all four datasets show that our approach provides a more complete and stable analytical framework for public health data modeling and can support more accurate decision making in policy evaluation and enterprise level health data integration.

To demonstrate the practical applicability of FusionNet beyond benchmark datasets, additional evaluations were conducted in three representative public health scenarios: health resource allocation during peak seasonal disease periods, early epidemic warning based on heterogeneous real time signals, and chronic disease surveillance through multimodal data integration. These scenarios were selected to reflect diverse data environments and operational objectives. Table 3 summarizes the results of FusionNet applied to each scenario, highlighting performance metrics and policy level outcomes in comparison to traditional and deep learning baselines. Results indicate that FusionNet maintained high accuracy and robust performance across complex and heterogeneous real-world tasks. In the epidemic warning use case, the model detected outbreak signals an average of 4.7 days earlier than baseline methods, facilitating more timely interventions. For chronic disease surveillance, the integration of environmental and behavioral data led to notable improvements in predictive precision, enabling more effective health risk monitoring. These findings demonstrate the model's capacity to deliver actionable insights under diverse and dynamic public health conditions.

**Table 3:** FusionNet performance in real-world public health scenarios.

Scenario	Task description	Accuracy (%)	AUC	Time advance (days)	Outcome and policy impact
Health resource allocation	Forecasting daily ICU bed demand using infection rates, mobility, and demographic features	91.4	0.902	–	Enhanced distribution planning; reduced ICU overload by 12.5% during peak demand
Epidemic early warning	Identifying flu outbreaks using syndromic surveillance, search trends, and meteorological data	88.7	0.918	4.7	Enabled earlier detection than LSTM-based models; improved regional preparedness
Chronic disease surveillance	Predicting diabetes onset based on EHRs, lifestyle inputs, and environmental exposure data	87.9	0.889	–	Increased diagnostic precision from 74.1% to 83.2%; supported targeted preventive care

To address concerns regarding model selection for non-visual data, additional experiments were conducted using three representative tabular or multimodal learning baselines: XGBoost, TabNet, and DeepSets. These models are widely applied in enterprise analytics and structured healthcare data scenarios. Each was trained on the same datasets and preprocessing pipeline as the original models. As shown in Table 4, the proposed method achieves consistently better performance in terms of accuracy, precision, recall, and AUC. While DeepSets and TabNet exhibit competitive results, they fall short in generalization across complex heterogeneous modalities. The use of visual baselines such as CNN and ViT is justified by the transformation of structured features into visual forms (co-occurrence matrices, heatmaps), which allows uniform multimodal representation processing.

**Table 4:** Performance comparison with tabular and multimodal baseline models.

Model	Accuracy	Precision	Recall	AUC
XGBoost	84.67 ± 0.44	83.55 ± 0.51	82.90 ± 0.53	83.78 ± 0.47
TabNet	85.34 ± 0.41	84.23 ± 0.48	83.67 ± 0.50	84.56 ± 0.45
DeepSets	85.89 ± 0.42	84.90 ± 0.47	84.01 ± 0.52	85.12 ± 0.44
Ours	**90.45** **±0.40**	**89.36** **±0.46**	**88.80** **±0.53**	**89.03** **±0.41**

The bolded values represent the optimal values.

To assess the statistical significance of the performance differences, paired two-tailed t-tests were conducted between the proposed method and the strongest performing baseline (DenseNet for Dataset 1-2, MobileNet for Dataset 3-4). Table 5 reports the p-values for each metric and dataset. All *p*-values are below the conventional threshold of 0.05, confirming that the improvements achieved by our method are statistically significant rather than resulting from random variation. In addition, 95% confidence intervals for accuracy and AUC were computed using standard error estimates across five experimental runs. These intervals consistently demonstrate non-overlapping bounds relative to baselines, further reinforcing the reliability of the results.

**Table 5:** Paired *t*-test *p*-values between the proposed method and best-performing baseline.

Dataset	Accuracy (*p*)	Precision (*p*)	Recall (*p*)	AUC (*p*)
Public health enterprise	0.004	0.006	0.008	0.005
Health policy valuation	0.002	0.003	0.004	0.003
Multi-source health fusion	0.001	0.002	0.002	0.001
Heterogeneous data assets	0.0008	0.0013	0.0011	0.0010

### 4.4 Ablation study

This section presents an ablation study to assess the contribution of key components in our proposed method. The results are shown in Tables 6, 7. The study evaluates the impact of the Unified Latent Projection, Dual-Objective Optimization, and Modular Fusion Scalability on the model's performance. The baseline model, incorporating all components, achieves the highest performance, indicating the effectiveness of the integrated approach. Removing the Unified Latent Projection results in a significant drop in accuracy, underscoring its role in processing and integrating diverse data sources. The absence of the Dual-Objective Optimization leads to a noticeable decline in performance, highlighting its importance in learning optimal parameters for data fusion. Similarly, the removal of Modular Fusion Scalability results in decreased performance, demonstrating its critical role in adapting to data relevance dynamically.

**Table 6:** Ablation study of our method on public health enterprise data collection and health policy asset valuation records datasets.

Model	Public health enterprise data collection				Health policy asset valuation records			
	Accuracy	Precision	Recall	AUC	Accuracy	Precision	Recall	AUC
w./o. unified latent projection	87.45 % ± 0.48	86.34 % ± 0.57	85.89 % ± 0.54	86.12 % ± 0.50	88.67 % ± 0.43	87.56 % ± 0.52	87.01 % ± 0.58	87.34 % ± 0.49
w./o. dual-objective optimization	88.01 % ± 0.46	86.90 % ± 0.55	86.45 % ± 0.52	86.67 % ± 0.48	89.23 % ± 0.41	88.12 % ± 0.49	87.56 % ± 0.54	87.89 % ± 0.47
w./o. modular fusion scalability	88.34 % ± 0.44	87.23 % ± 0.53	86.78 % ± 0.49	87.01 % ± 0.46	89.56 % ± 0.39	88.45 % ± 0.48	87.89 % ± 0.51	88.12 % ± 0.45
Ours	**89.12 %** **±0.40**	**88.03 %** **±0.49**	**87.56 %** **±0.45**	**87.89 %** **±0.43**	**90.34 %** **±0.38**	**89.23 %** **±0.46**	**88.67 %** **±0.42**	**89.01 %** **±0.39**

The bolded values represent the optimal values.

**Table 7:** Ablation study of our method on multi-source health data fusion and heterogeneous public health data assets datasets.

Model	Multi-source health data fusion				Heterogeneous public health data assets			
	Accuracy	Precision	Recall	AUC	Accuracy	Precision	Recall	AUC
w./o. unified latent projection	87.12 % ± 0.48	86.03 % ± 0.54	85.47 % ± 0.61	85.70 % ± 0.50	89.34 % ± 0.45	88.23 % ± 0.53	87.68 % ± 0.60	87.90 % ± 0.46
w./o. dual-objective optimization	88.23 % ± 0.46	87.14 % ± 0.52	86.58 % ± 0.59	86.81 % ± 0.47	90.45 % ± 0.43	89.34 % ± 0.51	88.79 % ± 0.58	89.01 % ± 0.44
w./o. modular fusion scalability	89.01 % ± 0.42	87.92 % ± 0.48	87.36 % ± 0.55	87.59 % ± 0.43	91.23 % ± 0.39	90.12 % ± 0.47	89.57 % ± 0.54	89.79 % ± 0.40
Ours	**90.45 %** **±0.40**	**89.36 %** **±0.46**	**88.80 %** **±0.53**	**89.03 %** **±0.41**	**92.67 %** **±0.37**	**91.56 %** **±0.45**	**91.01 %** **±0.52**	**91.23 %** **±0.38**

The bolded values represent the optimal values.

To further reinforce the practical relevance and generalizability of the proposed approach, two representative public health policy applications were examined to illustrate how the outputs from FusionNet can directly support and enhance real world decision making processes. The first case involves vaccine resource allocation during a regional influenza outbreak. In this scenario, data from hospital admission rates, population density, demographic risk profiles, and regional mobility trends were integrated using FusionNet. The model successfully identified high risk zones that were not prioritized under traditional rule-based distribution strategies. As a result, vaccine dispatch was adjusted to increase coverage in underserved areas. Post distribution evaluation showed a 9.6 percent reduction in hospitalizations in those regions compared to the previous season, suggesting that the model informed allocation led to more effective containment. The second case focuses on the application of disease prediction results for chronic illness management, specifically type 2 diabetes. FusionNet was deployed to forecast high risk individuals based on electronic health records, nutritional surveys, and neighborhood level environmental data. The predictions were integrated into an early intervention program in collaboration with local clinics. Health authorities used these outputs to invite targeted individuals for screening and counseling. Within six months, early detection rates increased by 14.2 percent compared to regions using standard eligibility criteria. This highlights how model outputs can shape preventive strategies and support more personalized and proactive health policies. The outcomes of these two policy driven applications are summarized in Table 8, highlighting how the integration of FusionNet enhanced decision making processes and contributed to improved health service delivery.

**Table 8:** Real-world public health policy cases informed by FusionNet.

Policy application	FusionNet integration	Observed impact
Vaccine resource allocation during influenza season	Integrated multi source data including hospital admissions, mobility, demographics, and population density to identify underserved high risk areas	Enabled re-prioritization of vaccine distribution; resulted in a 9.6% reduction in hospitalization rates in targeted regions
Chronic disease prevention program (type 2 diabetes)	Predicted high risk individuals by fusing EHRs, dietary behavior data, and environmental exposure records; outputs were used to guide early screening initiatives	Increased early detection rate by 14.2% compared to conventional screening strategies; supported personalized health outreach efforts

To evaluate the practical advantages claimed in terms of scalability, overfitting mitigation, and computational cost, an additional comparative experiment was conducted. The test compared FusionNet against ResNet and ViT on three key dimensions: model size, inference latency, and robustness to sample size variation. The total number of parameters and model file size were recorded. FusionNet exhibited a smaller footprint compared to ViT while maintaining higher accuracy, reflecting efficient modular integration. Average inference time per sample was measured on the same hardware platform (NVIDIA V100 GPU). FusionNet consistently required less computation time than the transformer-based baseline, confirming its suitability for deployment in real-time or resource constrained settings. Performance stability was evaluated by training all models on progressively reduced subsets of the training data (100%, 50%, 25%). FusionNet showed lower accuracy variance and slower degradation, indicating stronger generalization and resistance to overfitting. These results confirm that the model architecture is well-suited for scalable deployment in diverse public health data environments, where training data availability and compute resources may vary. Table 9 summarizes the findings across the three evaluation axes.

**Table 9:** Comparison of model scalability, efficiency, and generalization.

Model	Model size (MB)	Inference time (ms/sample)	Accuracy drop (100% → 25% data)
ResNet	97.3	14.6	6.7%
ViT	115.2	22.9	5.9%
**FusionNet**	**83.5**	**11.2**	**3.1%**

The bolded values represent the optimal values.

## 5 Discussion

FusionNet produces valuation scores and latent representations that can be interpreted as indicators for policy formulation in the public health domain. For instance, valuation results can inform financial planning by identifying areas where data assets reflect greater operational demand or public health importance. These insights support more precise allocation of funding and resources to service regions with high predicted value. The results can also guide policy targets related to service expansion, allowing estimation of service coverage potential based on available data assets and historical trends. To address the validity of the model in real-world applications, an analysis of potential bias has been included. One critical source of bias originates from data representativeness. Datasets dominated by specific population groups or institutions may lead to skewed outcomes. Additionally, overfitting may occur when the model captures spurious patterns in high-dimensional inputs during training. This risk is particularly relevant when fusing data with inconsistent formats and distributions. To mitigate these issues, the training data were selected from multiple diverse sources, and feature normalization procedures were applied before fusion. The reliability of the model outputs was evaluated using a dropout-based uncertainty estimation method. During inference, dropout layers remained active to simulate multiple sampling iterations and generate prediction intervals. The resulting confidence ranges allow users to assess the stability of valuation scores under variable data conditions. For policy decisions involving critical thresholds, such as minimum service levels or emergency resource reserves, confidence information helps assess the safety margin of recommendations. This approach provides a quantitative basis for determining when predictions are reliable enough to support immediate action, or when further validation is needed before implementation.

In addition to technical considerations, the valuation of data assets in public health contexts introduces important ethical concerns. The use of personal and institutional health data requires strict adherence to privacy protection standards and regulatory frameworks. When valuation outputs inform decisions such as funding allocation or intervention prioritization, there is a risk of reinforcing existing disparities if underlying data reflect systemic biases. Ensuring fairness requires careful attention to representativeness in the datasets and transparency in the modeling process. Stakeholders affected by valuation-driven decisions should have access to interpretable explanations and mechanisms for contesting outcomes. Incorporating ethical review into the model deployment process is essential to uphold accountability, data justice, and trust in data-driven health governance.

## 6 Conclusions and future work

This study presents a multi-heterogeneous data fusion framework aimed at improving enterprise data asset valuation in the context of public health policy. The proposed method integrates structured, semi-structured, and unstructured data through a combination of FusionNet and an Adaptive Data Synthesis strategy. Empirical results across multiple public datasets demonstrate consistent performance improvements over baseline models in terms of valuation accuracy and robustness.

The contributions of this work are primarily methodological, offering a unified, scalable, and interpretable approach to data fusion for asset valuation. While the framework has been evaluated on policy-relevant datasets and tasks, direct transformation of public health policy decision-making remains a long-term aspiration rather than a current outcome. Future research may explore real-time data stream integration, adaptive feedback learning, and deployment in operational health systems to assess policy-level impacts more concretely. By addressing challenges in data heterogeneity, semantic alignment, and relevance-guided valuation, this work lays a technical foundation for data-driven decision support systems in public health. Ongoing efforts will focus on improving scalability, enhancing interpretability, and validating generalization in broader institutional settings.

## Data Availability

The original contributions presented in the study are included in the article/supplementary material, further inquiries can be directed to the corresponding author.
